# Effect of K/Al Molar Ratio on the Thermo-Mechanical Properties of Metakaolinite-Based Geopolymer Composites

**DOI:** 10.3390/polym13213754

**Published:** 2021-10-29

**Authors:** Jan Kohout, Petr Koutník, Pavlína Hájková, Eliška Kohoutová, Aleš Soukup

**Affiliations:** 1 ORLEN UniCRE a.s., Revoluční 1521/84, 400 01 Ústí nad Labem, Czech Republic; petr.koutnik@unicre.cz (P.K.); Pavlina.Hajkova@unicre.cz (P.H.); eliska.haincova@unicre.cz (E.K.); Ales.Soukup@unicre.cz (A.S.); 2 Department of Material Science, Faculty of Mechanical Engineering, Technical University of Liberec, Studentská 1402/2, 461 17 Liberec, Czech Republic

**Keywords:** K-geopolymer, metakaolinite, claystone, thermal properties, mechanical properties

## Abstract

A metakaolinite-based geopolymer binder was prepared by using calcined claystone as the main raw material and potassium as the alkaline activator. Chamotte was added (65 vol%) to form geopolymer composites. Potassium hydroxide (KOH) was used to adjust the molar ratio of K/Al and the effect of K/Al on thermo-mechanical properties of geopolymer composites was investigated. This study aimed to analyze the effect of K/Al ratio and exposure to high temperatures (up to 1200 °C) on the compressive and flexural strengths, phase composition, pore size distribution, and thermal dilatation. With an increasing K/Al ratio, the crystallization temperature of the new phases (leucite and kalsilite) decreased. Increasing content of K/Al led to a decline in the onset temperature of the major shrinkage. The average pore size slightly increased with increasing K/Al ratio at laboratory temperature. Mechanical properties of geopolymer composites showed degradation with the increase of the K/Al ratio. The exception was the local maximum at a K/Al ratio equal to one. The results showed that the compressive strength decreases with increasing temperature. For thermal applications above 600 °C, it is better to use samples with lower K/Al ratios (0.55 or 0.70).

## 1. Introduction

Geopolymers are inorganic materials with a wide variety of applications and superior properties which have been intensively studied over the past five decades [1,2,3]. Geopolymers attract attention due to their excellent mechanical properties, high temperature resistance (geopolymers do not ignite, burn, or release any smoke during exposure to fire), and resistance to chemicals (mainly acids and organic solvents) [4,5,6]. Numerous applications of geopolymers have been developed based on their properties—building materials [7], coatings [8], catalysts [9], fiber composites [10,11], sorbents [12], materials for 3D printing [13], waste immobilization [14], etc. Various inert solid materials (quartz, chamotte, cordierite or corundum, etc.) are usually used as a filler in order to improve the properties and/or reduce the price of geopolymer binders [15,16]. Special application of geopolymer composites is a replacement for expensive ceramic materials as heat-resistant materials [17].

Geopolymers are synthesized by mixing powdered aluminosilicates with a liquid alkaline activator at a laboratory (LT) or slightly higher temperatures. Typically used alkaline activators are aqueous alkali metal hydroxide or alkali silicate (water glass). The most commonly used aluminosilicates are metakaolin (calcined kaolin), fly ash, volcanic ash, blast furnace slag, demolition wastes, or rice husk ash [3,18,19]. The process of geopolymer synthesis involves partial dissolution of solid material rich in Al and Si in an alkaline medium, followed by polycondensation reactions of hydrolyzed silicates and aluminates into a three-dimensional polymer network [20,21].

Geopolymer properties depend on the type of aluminosilicate [1,22], the type of alkali cation (Na^+^ or K^+^) [23], molar ratios (Si/Al, Si/Me, Me/Al; Me marks an alkali metal) [24,25,26], water content [7,27], curing conditions [28], and types of fillers [2,15]. The effect of Si/Al molar ratios on the mechanical properties and microstructure of geopolymers were investigated in many studies [24,29,30]. Better mechanical properties have been reported for mixtures with Si/Al ratios in the range of 1.5–1.9 [25,31]. The Me/Al molar ratio has been studied primarily with sodium cation [32,33]. Subaer et al. found that with increasing Na/Al molar ratio, the compressive strength of geopolymer binders decreased. Other studies discovered that decreasing the Na/Al molar ratio led to the pore sizes of geopolymer binders increasing and that the highest compressive strengths can be attained when the Na/Al ratio is close to one [33,34]. Reaction rate and extent in all geopolymerization processes can be accelerated and increased by an increased Na/Al ratio [32].

It was found that the potassium-based geopolymers have better compressive strengths than sodium-based geopolymers [35]. The molar ratio of K/Al also have a significant influence on the properties of the geopolymer, but so far, nearly no research has been carried out on discovering the effect of K/Al molar ratio on the thermo-mechanical properties of geopolymers. Tawfik et al. [36] investigated the effect of the K/Al molar ratio between 0.9–1.55 and type of curing on the microstructure and phase formation of a metakaolinite-based geopolymer. They discovered that increasing the molar ratio of K/Al had no significant effect on phase composition of the geopolymer binder.

The aim of this study was to investigate the effect of K/Al molar ratio on the thermo-mechanical properties of metakaolinite-based geopolymer binder/chamotte composites. The results were compared with the properties of the unfilled geopolymer binders. Geopolymer binders were prepared from calcined kaolinitic claystone and a potassium alkaline activator.

## 2. Materials and Methods

### 2.1. Materials

**Figure 1:**
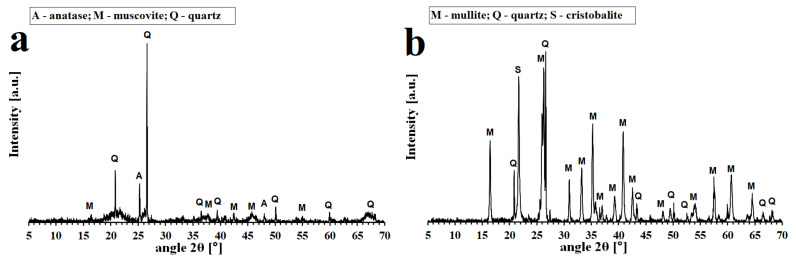
XRD patterns of the aluminosilicate Mefisto L_05_ (**a**) and chamotte (**b**).

Starting materials used for the preparation of geopolymer binders were commercial metakaolinite-rich material Mefisto L_05_ produced by the calcination of kaolinitic claystone at about 750 °C in a rotary kiln (České lupkové závody, a.s., Nové Strašecí, Czech Republic), potassium silicate (specific gravity 1384 kg/m^3^, Vodní sklo, a.s., Prague, Czech Republic) and potassium hydroxide pellets (G.R. grade, 88.2 wt% KOH, Lach-Ner, s.r.o., Neratovice, Czech Republic). Chamotte (České lupkové závody, a.s., Nové Strašecí, Czech Republic) of grain size 0–2 mm was used as a natural filler for the preparation of the geopolymer composites. The chemical compositions of the raw materials are given in Table 1, physical properties are summarized in Table 2, and the results of X-Ray diffraction analysis (XRD, Bruker, Billeica, MA, USA) of aluminosilicate Mefisto L_05_ and chamotte are shown in Figure 1.

### 2.2. Preparation of Geopolymers

The alkali activators were obtained by dissolving the solid potassium hydroxide in a commercial potassium silicate solution so that the K:Si molar ratios were 1.2, 1.5, 1.8, 2.1, 2.4, and 2.7. The metakaolinite component Mefisto L_05_ was dried at 110 °C for 24 h. The geopolymer binders were prepared by mixing dried metakaolinite with alkali activators in a planetary mixer at room temperature for 10 min. Distilled water was then added to achieve a total water content of 30% in the geopolymer binder and mixing was continued for another 5 min. The homogenous mixtures were placed into silicon moulds and vibrated for 5 min in order to remove any air bubbles. The moulds with prepared samples were sealed into polyethylene bags and cured at 60 °C for 4 h in an electric oven. The samples were de-moulded and left to cure at laboratory temperature (LT, 20 °C) for 7 days. The curing conditions, including time, were chosen on the basis of the results of Rovnaník et al. [28], which verified that, under these conditions, the samples reached final strengths.

The prepared geopolymer binders had molar ratios of K:Al = 0.55, 0.70, 0.85, 1.0, 1.15, and 1.30. The Si:Al molar ratio was 1.5 for all tested geopolymer binders. The total water content was 30% in the case of all prepared geopolymer binders. Total water content involved water contained in the commercial potassium silicate solution and potassium hydroxide together with the water added during the binder preparation. The geopolymer binders were chosen on the basis of the results of our previous works, where the geopolymer binders based on Mefisto L_05_ provided binders with very low viscosity and excellent mechanical properties [19,37]. The geopolymer binders were labelled as GB-X, where X indicates molar ratio of Me:Al. 

Geopolymer composites containing 65 vol% of granular inorganic filler were prepared by adding chamotte to pure geopolymer binder (GB) and mixing for another 5 min. The curing conditions were the same as for the geopolymer binders without chamotte addition. The addition of filler was chosen according to the results of our previous work, where the geopolymer composites filled with chamotte provided the optimal properties [15]. The composites with chamotte admixture were labelled as GS-X.

Samples of geopolymer binders and geopolymer composites were cured for 7 days as described above, and were exposed to heat at 200, 400, 600, 800, 1000, and 1200 °C. The samples were placed into an electric furnace (Clasic, type 5013V, Řevnice, Czech Republic) and heated at a rate of 5 °C/min, and heat saturated for 1 h at each desired temperature. Subsequently, the samples were cooled inside the furnace down to laboratory temperature. The samples were labelled as GB(S)-X-Y, where Y indicated the temperature exposure in °C.

### 2.3. Analytical and Testing Methods

Particle size distribution of the aluminosilicate Mefisto L_05_ was measured by a Mastersizer 2000 laser diffraction particle size analyzer (MALVERN Instruments, Malvern, UK). Agglomerates were disrupted by an ultrasound treatment.

A pycnometric method was used to determine specific gravity and bulk density by pouring powder into a cylindrical vessel.

Brunauer–Emmett–Teller (BET) surface area of the aluminosilicate Mefisto L_05_ was determined by nitrogen adsorption using an Autosorb iQ (Quantochrome Instruments, Boynton Beach, FL, USA).

Chemical compositions of the solid raw materials were determined by X-ray fluorescence (XRF, Bruker, Billerica, MA, USA) using a BRUKER S8 Tiger instrument.

An inductively coupled plasma optical emission spectrometer (ICP-OES) OPTIMA 8000 (Perkin Elmer, Waltham, MA, USA) was used to determine the content of micro-elements and the K/Na ratio in the liquid potassium silicate. Conventional acid-base titration methods were used for determining total content of alkali metals (Na, K) and SiO_2_ in the potassium silicate (module) solutions. These methods were preferred for their better accuracy at higher concentrations. 

An AutoPore IV 9510 mercury intrusion porosimeter (Micromeritics, Unterschleißheim, Germany) was used to determine the pore size distributions of geopolymer composites before and after exposure to a high temperature up to 1200 °C. The porosimeter operates with pressures from 0.01 MPa to 414 MPa. Pore size distribution was evaluated in the range of 4 nm–350 μm for geopolymer binders and composites (full measurement range).

The phase composition of the raw materials, geopolymer binders, and composites was identified by means of a BRUKER D8 Advanced X-Ray diffraction system (XRD) equipped with a BRUKER SSD 160 detector and operating with Cu-Kα radiation with an X-ray source at 40 kV and 25 mA. XRD scanning was performed at the step 2θ = 0.02° over an angular range from 5° to 70° with 1 s dwell time.

Dilation tests up to 1200 °C using a dilatometer (Clasic CZ, type DIL 1500, Řevnice, Czech Republic) were carried out on 5 mm × 5 mm × 50 mm samples of geopolymer binders and 20 mm × 20 mm × 160 mm of geopolymer composites. The measurements were conducted under the same heating conditions (5 °C/min) for all the samples.

A heating microscope for the determination of pyrometric cone refractoriness (Clasic CZ, type 0116 VAK, Řevnice, Czech Republic) of geopolymer binders and geopolymer composites was used. Pyrometric cone refractoriness was determined according to the European standard EN 993-12. This test was carried out with a sharp-edged, trilateral, oblique, truncated cone with the dimensions of 30 mm × 8.5 mm. The temperature was increased at a rate of 5 °C/min. The pyrometric cone refractoriness was determined by simultaneously melting two reference cones with different melting points and placing the tested cone between them.

A universal testing machine, LabTest 6.200 (Labortech, Opava, Czech Republic) fitted with an electric furnace, allowing testing of mechanical properties at temperatures elevated up to 1200 °C, was used for the determination of mechanical properties. Flexural strength was determined using a three-point-bending test on six samples (20 mm × 20 mm × 160 mm) of geopolymer composites before and after exposure to high temperatures up to 1200 °C with a crosshead speed of 0.1 MPa/s (approx. 0.25 mm/min). Compressive strength and modulus of elasticity were measured according to the ISO 1920-10 standard on six prismatic samples (30 mm × 30 mm × 64 mm) of geopolymer composites before and after exposure to high temperatures up to 1200 °C with a crosshead speed of 0.5 MPa/s (approx. 0.25 mm/min). The compressive strength and modulus of elasticity of the geopolymer composites measured in situ at temperatures from 25 to 1200 °C were also tested. The compressive strength and modulus of elasticity of the geopolymer composites were measured on the three prismatic samples (30 mm × 30 mm × 64 mm). The temperature was increased at a rate of 5 °C/min and lasted 1 h at each examined temperature. Mechanical properties were determined 7 days after the sample preparation.

Morphology of the geopolymer composites was studied by a scanning electron microscope JSM-IT500HR from JEOL (JEOL, Tokyo, Japan). Samples were coated with about 5 nm of gold to make them conductive and to prevent charging of the samples in the microscope.

## 3. Results and Discussion

### 3.1. Phase Composition

**Figure 2:**
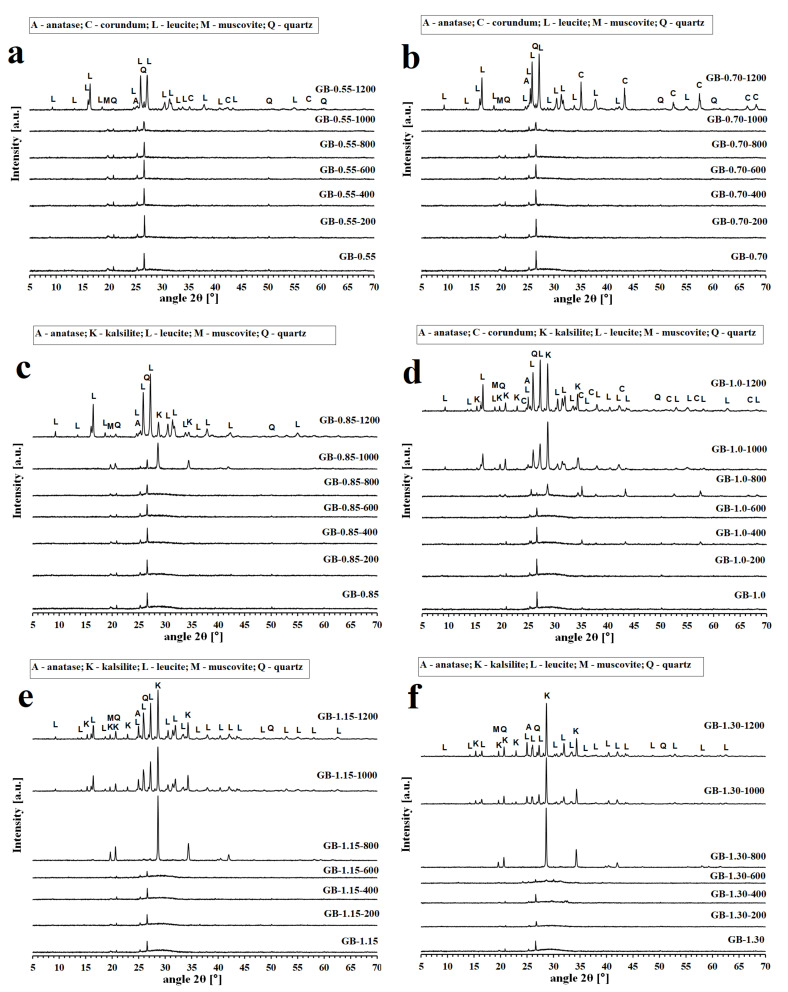
XRD patterns of the geopolymer binders with various molar ratios of K/Al ((**a**)—GB-0.55, (**b**)—GB-0.70, (**c**)—GB-0.85, (**d**)—-1.0, (**e**)—GB-1.15, (**f**)—GB-1.30) at laboratory temperature and after exposure up to 1200 °C.

**Figure 3:**
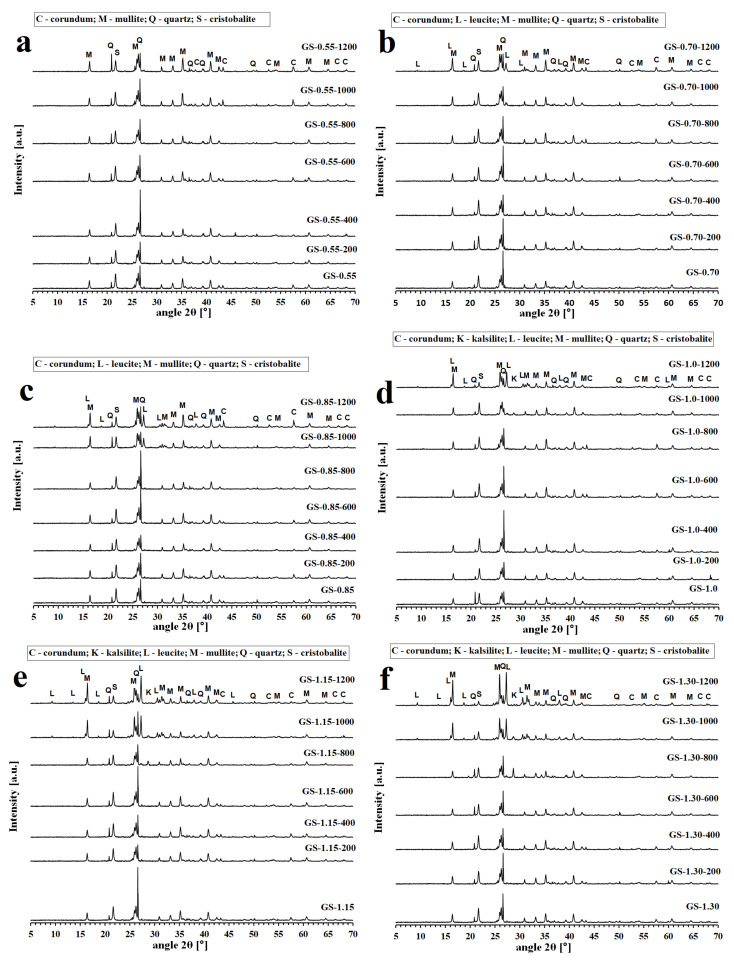
XRD patterns of the geopolymer composites with various molar ratios of K/Al ((**a**)—GS-0.55, (**b**)—-0.70, (**c**)—GS-0.85, (**d**)—GS-1.0, (**e**)—GS-1.15, (**f**)—GS-1.30) at laboratory temperature and after exposure up to 1200 °C.

The X-ray diffraction patterns of the geopolymer binders and composites with various molar ratios of K/Al tested before and after exposure to temperatures up to 1200 °C are reproduced in Figure 2 and Figure 3. The impurities of corundum (Al_2_O_3_) identified in some of examined samples were caused by milling in a corundum mill with corundum balls.

On heating, the X-ray pattern for geopolymer binders (Figure 2) remained predominantly amorphous until about 800 °C. The XRD patterns of the geopolymer binders revealed that the presence of leucite (KAlSi_2_O_6_) and kalsilite (KAlSiO_4_) as the main crystalline phases occurred from 800 °C to 1200 °C depending on increasing K/Al. With increasing content of alkalis (potassium), there was an earlier (at lower temperature) formation of crystalline phases. For example, in the sample GB-0.55, the crystalline phase was visible only at a temperature equal to 1200 °C. On the contrary, in the sample GB-1.30, the crystalline phases were visible already at a temperature equal to 800 °C. The kalsilite content increased with increasing ratios of K/Al. Leucite and kalsilite are common crystalline phases for potassium geopolymer binders formed after heat exposure at temperatures ranging from 800 °C to 1300 °C [36,38,39,40,41]. Crystalline impurities such as quartz (SiO_2_), anatase (TiO_2_), and muscovite (K(Al_4_Si_2_O_9_(OH)_3_)) were present in all geopolymer binders. These impurities originated from the raw materials (Table 2 and Figure 1).

Similar changes in XRD patterns were obtained in the case of geopolymer composites (Figure 3). Kalsilite and leucite crystalline phases formed at high temperatures from the amorphous matrix. The formation of crystalline phases at lower temperatures was observed with geopolymer composites with increasing K/Al ratios as in the case of geopolymer binders. In the sample of GS-1.30, the crystalline phases were already visible after exposure to 800 °C. However, no crystalline phases were visible in the GS-0.55 sample even after exposure to 1200 ° C. The low intensity of leucite could be shielded by the high intensity of mullite. The results showed that the chamotte remained inert and did not react with the geopolymer matrix at high temperatures. The presence of the crystalline phase of quartz (SiO_2_) and its high-temperature form of cristobalite in geopolymer composites is due to the fact that quartz is present as an impurity in the starting aluminosilicate raw material and in the chamotte filler (as cristobalite). Mullite (Al_6_Si_2_O_13_) is the main component of chamotte, as shown in Figure 1b.

### 3.2. Thermal Properties

#### 3.2.1. Thermal Dilatometry

**Figure 4:**
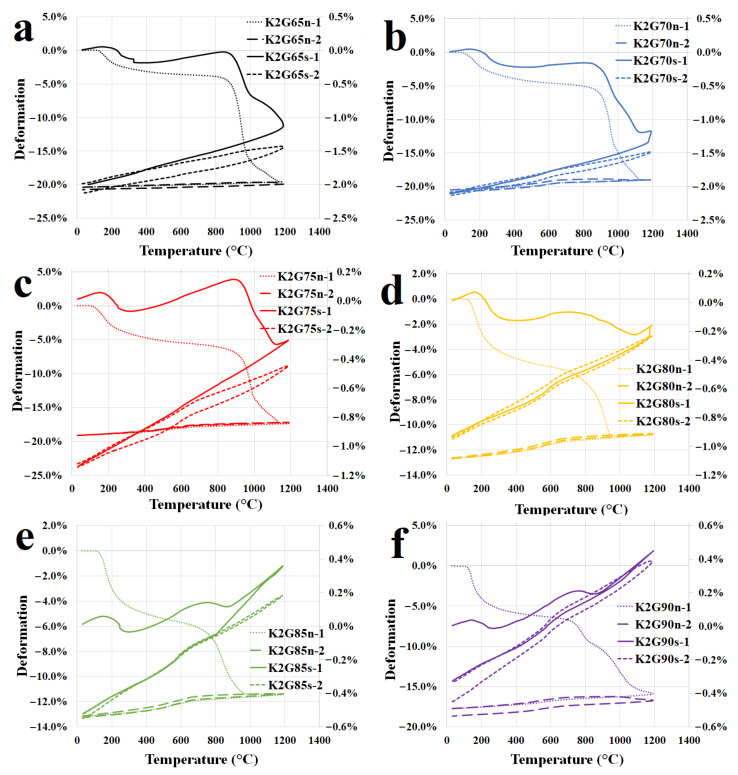
The dilatometric curves of the first and second runs of geopolymer binders (y-axis on the left) and composites (y-axis on the right) with various molar ratios of K/Al ((**a**)—GB-0.55 and GS-0.55, (**b**)—GB-0.70 and GS-0.70, (**c**)—GB-0.85 and GS-0.85, (**d**)—GB-1.0 and GS-1.0, (**e**)—GB-1.15 and GS-1.15, (**f**)—GB-1.30 and GS-1.30) up to 1200 °C (5 °C/min).

Figure 4 shows the dilatometric curves (first and second run) of the geopolymer binders with various K/Al molar ratios recorded within the range from 30 °C to 1200 °C with a heating rate of 5 °C/min. The dilatometric curves showed that all samples of geopolymer binders shrank in the temperature range between 150 °C to 700–900 °C (depending on the K/Al ratio). Initial shrinkage was due to the dehydration of free evaporable water from the pores at temperatures under 300 °C. In the temperature range of 300 to 700–900 °C, continued shrinkage was observed in all samples due to dehydroxylation. The second major shrinkage occurred at a temperature of 700–900 °C (depending on the sample) up to the temperature limit of the experiment (1200 °C). This was presumably caused by the crystallization of new phases (leucite and kalsilite) or by the dissolution of undissolved metakaolinite. The onset temperatures of the major shrinkage (crystallization) decreased with increasing K/Al ratios, which is in accordance with the XRD patterns described above (Figure 2). Significant shrinkage of the sample GB-0.55 was observed at a temperature of about 900 °C and in the case of the sample GB-1.30 at 700 °C. Inconspicuous linear shrinkage was observed at all samples of geopolymer binders during cooling. The geopolymer binders expanded linearly during the second run of dilatometry. The heating and cooling curves in the second run of dilatometry copied the cooling curve in the first run. This fact has already been observed by Lemougna et al. [26]. It can be observed that total shrinkage of geopolymer binders was in the range of 12–20% from their total length after the first run of heating and cooling. The smallest total shrinkage (about 12%) was found with samples GB-1.0 and GB-1.15. This finding corresponds well with the previous research in which the potassium geopolymer binders experienced similar total shrinkage—Kuenezel et al. [42] (25%), Kovařík et al. [43] (15%), and Lahoti et al. [29] (12%). The linear coefficients of thermal expansion (α) of samples GB-0.55, GB-0.70, GB-0.85, GB-1.0, GB-1.15, and GB-1.30 were about 0.82, 2.55, 2.59, 2.61, 2.55, and 2.39 × 10^−5^/°C. These coefficients α were measured between 200 and 600 °C during the second run of heating.

Dilatations (first and second run) of geopolymer composites with different K/Al molar ratios are displayed in Figure 4. In the first run of dilatation, all experimental samples of geopolymer composites exhibited a small expansion in the temperature range from 30 to 150 °C. This change was probably caused by the expansion of the fillers. The mildly expanding area within the temperature range from 150 °C to about 700–900 °C (depending on the type of sample) is given by the compensation of the binder shrinkage due to dehydroxylation and filler expansion. A leading change was observed with temperatures between 700–900 °C (depending on K/Al) as well as in the case of geopolymer binders. Significant shrinkage occurred in the case of samples GS-0.55, GS-0.70, GS-0.85, slight shrinkage for sample GS-1.0, and more significant expansion in the case of samples GS-1.15 and GS-1.30. These changes were due to a combination of new phases crystallization (kalsilite and leucite) and filler expansion. The temperature limit of the onset of crystallization decreased as the potassium content increased and at the same time, the tested samples expanded remarkably. Thermal changes of the filler (chamotte) correspond to its linear coefficient of thermal expansion (α), which is approximately 4.5 × 10^–6^ /°C, and the value was measured in the temperature range from 25 °C to 900 °C [44]. Chamotte, used as a filler, is a heat-resistant material, so the geopolymer composites that contain it are not so prone to changes in volume. Therefore, it is evident from Figure 4 that the presence of chamotte in all geopolymer binders significantly reduced shrinkage. Total shrinkage of all geopolymer composites did not outreach 2.1% of their total length after the first run of heating and cooling. In the research papers of Rovnaník et al. [1] and Kovařík et al. [43], the total shrinkage of potassium geopolymer composites with chamotte also did not exceed 2.5%. The samples of GS-0.85 and GS-1.0 had optimal behavior under the first run of heating, the samples of GS-0.55 and GS-0.70 showed significant shrinkage, and GS-1.15 and GS-1.30 samples had obvious expansion. The linear coefficients of thermal expansion (α) of GS-0.55, GS-0.70, GS-0.85, GS-1.0, GS-1.15, and GS-1.30 were 4.96, 5.13, 5.89, 6.14, 6.21, and 6.96 × 10^−6^/°C. These values were measured between 200 and 1000 °C during the second run of heating. The coefficients of thermal expansion indicated that thermal expansion increased with increasing alkali content.

Low shrinkage of geopolymer composites proves the possibility of their use in thermal engineering (thermally stressed applications, linings in chimneys, or fireplaces).

#### 3.2.2. Pyrometric Cone Refractoriness

**Figure 5:**
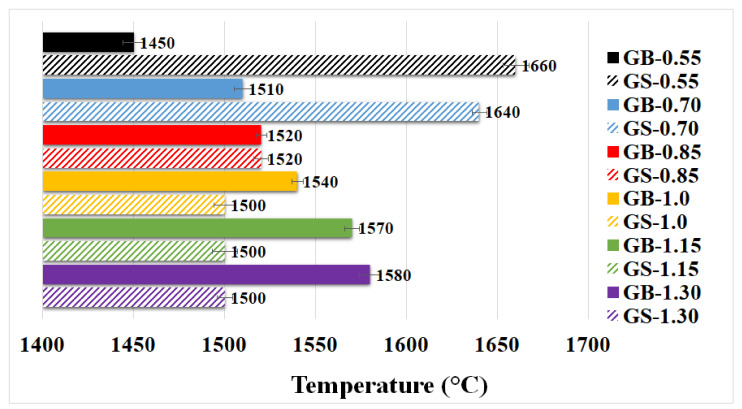
Pyrometric cone refractoriness of geopolymer binders (GB) and composites (GS) with various molar ratios of K/Al.

The ability to resist high temperatures (refractoriness) was examined for geopolymer binders and composites with various K/Al molar ratios. The results of these tests are shown in Figure 5. The refractoriness of geopolymer binders increased with increasing K/Al. The temperature difference between the limit samples (GB-0.55 and GB-1.30) was 130 °C. The addition of chamotte aggregate to the geopolymer binders resulted in a decrease in their refractoriness. The reduction in refractory value could be due to earlier sintering of the geopolymer matrix and chamotte or due to the lower melting point of the chamotte filler. The exceptions were samples GS-0.55 (1660 °C) and GS-0.70 (1640 °C), which had a refractory temperature significantly higher than the corresponding geopolymer binders GB-0.55 (1450 °C) and GB-0.70 (1510 °C). These two samples also differed from other geopolymer composites, which had a refractory temperature of around 1500 °C. This phenomenon can be explained by the fact that the samples firstly began to collapse inwardly, instead of starting to fall with the tip of the cone to the base. High values of refractoriness may not be due to high temperature resistance of these samples. The results reveal that almost all prepared samples (except GB-0.55) could withstand high temperatures up to 1500 °C. This was especially true of samples with a K/Al ratio equal to 0.85 or 1.0. The result of refractoriness of GS-1.0 is comparable with the result (1500 °C) of our previous research [15], where GS-1.0 was also tested.

Pyrometric cone refractoriness confirms the previously found information that the prepared geopolymer composites achieve very good heat resistance. The samples are well suited for use in high-temperature applications as a replacement for expensive ceramic materials.

### 3.3. Mechanical Properties

**Figure 6:**
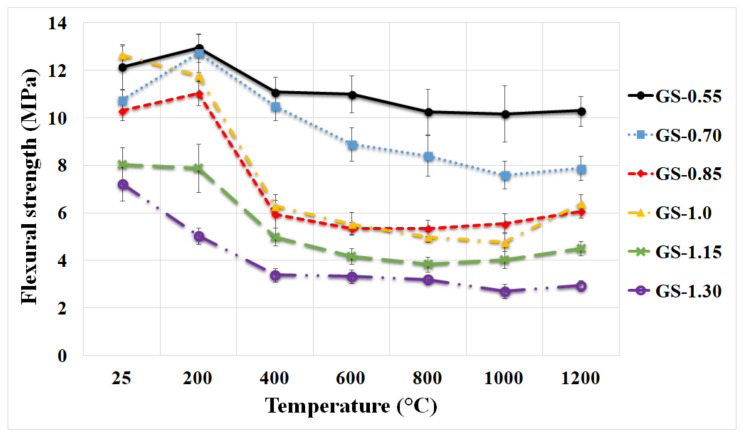
Flexural strength of the geopolymer composites with various molar ratios of K/Al at laboratory temperature and after exposure up to 1200 °C.

Figure 6 shows the flexural strength of all prepared geopolymer composites with various K/Al ratios before and after exposure to high temperatures. It can be seen that the values of flexural strength of geopolymer composites at laboratory temperature and after heat exposure to 200 °C were comparable (around 12 MPa) with increasing K/Al ratios up to a value of 1, followed by a significant decrease in strength with increasing K/Al ratios. Okada et al. observed a similar trend in the flexural strength curves with increasing Na/Al for metakaolin-based geopolymer binders [45]. Higher temperatures (up to 1200 °C) and higher potassium content (up to 1.30) resulted in a decrease of flexural strengths of geopolymer composites. The decrease of flexural strength at high temperatures for samples with higher K/Al ratios was probably due to their earlier crystallization (see Figure 3). Changes in flexural strengths of geopolymer composites after exposure to high temperatures followed a decreasing trend up to 1000 °C. The causes of the decrease were probably dehydration and the formation of cracks in the material during heating and cooling [43,46]. Exposure to 1200 °C led to a slight increase in flexural strength, which can be explained by the start of sintering of the material. A decline in flexural strength increases due to thermal exposure with increasing K/Al molar ratios.

The samples GS-1.0 and GS-0.55 showed the highest flexural strength (12.7 and 12.2 MPa) at laboratory temperature. The samples GS-0.55 and GS-0.70 had a flexural strength of 10.3 and 6.4 MPa after exposure to 1200 °C. The sample GS-0.55 excelled in great stability because the decrease in flexural strength was only 15% between LT and 1200 °C. On the contrary, a higher decrease in flexural strength (50%) between LT and 1200 °C was observed for the GS-1.0 sample. The sample GS-1.30 had flexural strengths of only 7.2 and 2.9 MPa at laboratory temperature and after exposure to 1200 °C. Musil et al. [47] also noticed a decrease in flexural strength with increasing temperature (LT—13 MPa and 1200 °C—3.44 MPa) for potassium geopolymer with addition of chamotte as a filler.

**Figure 7:**
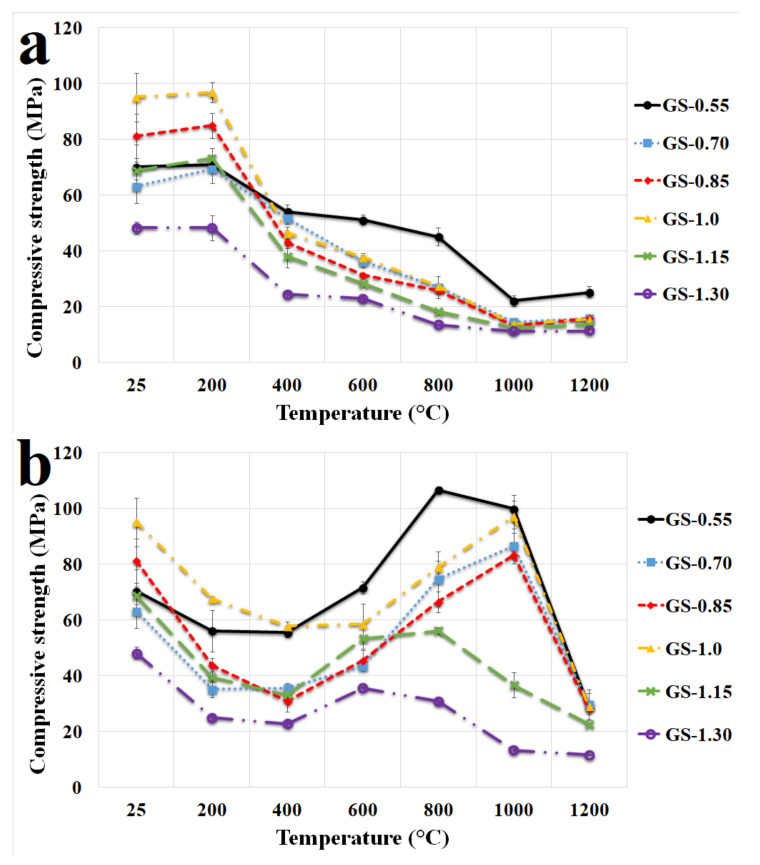
Compressive strength of the geopolymer composites with various molar ratios of K/Al at laboratory temperature and after exposure up to 1200 °C (**a**) and in situ temperature from 25 °C to 1200 °C (**b**).

Compressive strengths of geopolymer composites with varied K/Al before and after exposure to high temperatures are given in Figure 7a. The results confirmed the earlier published finding, i.e., a decrease in compressive strength with increasing temperatures (from average 70 MPa to about 15 MPa at 1000 °C) in geopolymer composites (the exceptions were some samples after exposure to 200 °C) [1,48,49,50]. The decrease in compressive strength was caused by dehydration of the geopolymer matrix and possible simultaneous development of microcracks and internal damage in the geopolymer structure [46]. A minor increase in compressive strength was observed in all samples after exposure to 200 °C. This change can be attributed to the solidification of the gel and increase in surface forces between the particles due to the release of adsorbed moisture. The compressive strength of the geopolymer composites also increased after exposure to 1200 °C. The increase could probably be due to sintering of the samples as in the case of flexural strength. Compressive strength decreased with increasing K/Al ratio. The exception was the local maximum at K/Al ratio equal to one, which was significant at laboratory temperature and after exposure to 200 °C. The local maximum decreased with higher exposure temperatures and at 1000 °C and 1200 °C was no longer evident. The trend of increase and consecutive decrease in compressive strength with increasing Na/Al ratios was observed by Liu et al. [33]. They achieved the highest compressive strength with a sample with Na/Al equal to one. A moderate decrease in compressive strength was noted with tested geopolymer composites with an increased potassium content at higher temperatures (from 400 °C to 1200 °C).

When the K/Al ratio was 1.0, the compressive strength of GS-1.0 sample reached the maximum (95.2 MPa) at laboratory temperature. Almost all examined geopolymer composites had comparable results of compressive strength (approx. 14 Mpa) after calcination to 1200 °C. An exception was the sample GS-0.55, which had a compressive strength of 25.2 MPa. Measured results of compressive strength of geopolymer composites with chamotte at laboratory temperature and after exposure to higher temperature were higher compared with previously published results by Rovnaník et al. [1,49] (37 MPa at 25 °C and 10 MPa at 1200 °C) and Trindadea et al. [48] (62 MPa at 25 °C and 18 MPa at 1200 °C). Higher values of compressive strength can be attributed to the appropriate selection of optimized geopolymer binders based on calcined claystone, which were previously verified by Koutník et al. [19].

Figure 7b displays the compressive strength of geopolymer composites with varied K/Al measured at temperatures from 25 °C to 1200 °C (in situ). It is evident that compressive strength tested in situ at the required temperature did not show results comparable with compressive strength tested after exposure to an elevated temperature (via Figure 7a). A decrease in compressive strength up to a temperature of 400 °C was also observed with in situ-investigated samples; the drop was caused by the previously mentioned dehydration. From temperatures of 600 to 1000 °C, the samples tested in situ had significantly higher values of compressive strength compared with samples tested after exposure to an elevated temperature. The increase in compressive strength was probably due to the absence of a cooling phase during in situ testing, which can be a source of cracks and increasing plasticity of the geopolymer composite at higher temperatures [15]. Sample GS-1.0 had higher compressive strength values tested in situ at elevated temperatures compared with samples GS-0.70, GS-0.85, GS-1.15, and GS-1.30. Sample GS-0.55 had analogous results to sample GS-1.0. Samples GS-0.55 and GS-1.0 gave exceptional results of compressive strengths (99.9 and 97.1 MPa) at an elevated temperature of 1000 °C.

**Figure 8:**
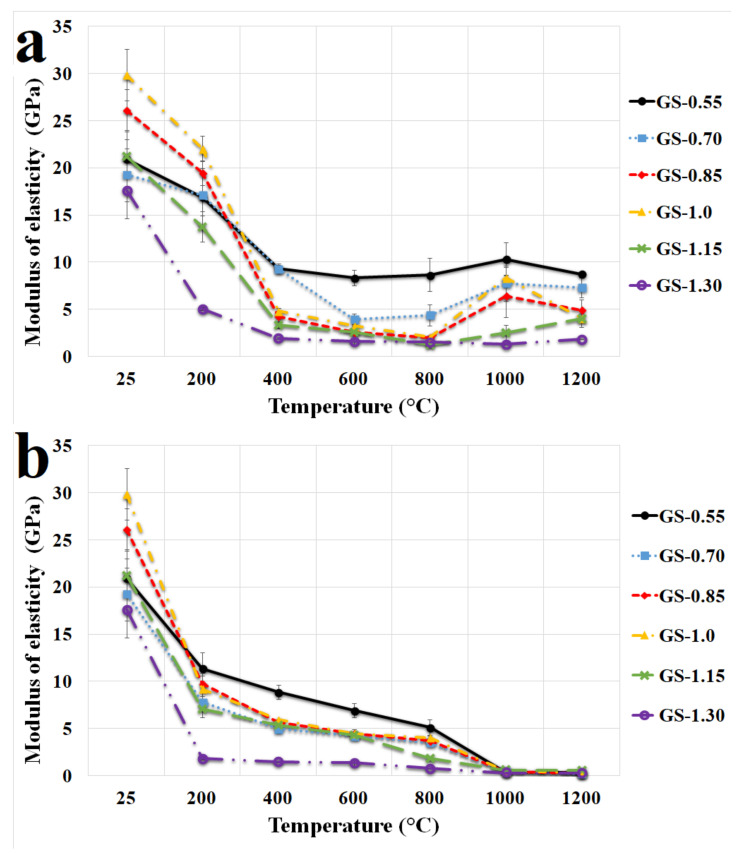
Modulus of elasticity of the geopolymer composites with various molar ratios of K/Al at laboratory temperature and after exposure up to 1200 °C (**a**) and in situ temperature from 25 °C to 1200 °C (**b**).

Figure 8a shows the dependences of the modulus of elasticity of geopolymer composites on the K/Al ratio in the binder for different exposure temperatures (ex situ). Curves of modulus of elasticity at different temperatures with different K/Al ratios were very similar to curves of compressive strength (Figure 7a), which meant a reduction in the modulus of elasticity with higher exposure temperatures and analogous behavior depending on the different K/Al ratios. A significant decrease in the modulus of elasticity was observed from a temperature of 200 °C due to dehydration of water from the pores of geopolymer binders. The values of modulus of elasticity first increased and then decreased with rising K/Al ratios. Similar behavior was observed by Zhang et al. [51], who investigated the geopolymer binders with different Si/Al and Na/Al molar ratios prepared from metakaolin. GS-1.0 had a modulus of elasticity 29.9 GPa at laboratory temperature and 4.1 GPa after exposure to 1200 °C. The highest modulus of elasticity after exposure to 1200 °C was with sample GS-0.55, and the value was 8.8 GPa. The modulus of elasticity observed by Zhang et al. [51] was 1 GPa with the sample with an Na/Al ratio equal to one tested at laboratory temperature. The large difference in modulus of elasticity may be due to different used cation, curing conditions, or higher water content.

The modulus of elasticity of geopolymer composites with varied K/Al tested in situ at elevated temperatures up to 1200 °C are given in Figure 8b. It is clear from the results that the modulus of elasticity tested in situ decreased with elevated temperature as well as in the case of samples tested after heat exposure and cooling (via Figure 8a). The modulus of elasticity of all tested samples was almost zero after exposure to 1000 °C and 1200 °C due to the plastic behavior of geopolymer binders at higher temperatures (1000 and 1200 °C). The dependence of the deformation on the stress was nonlinear in terms of a high increase in deformation. The plastic behavior was probably caused by the already mentioned new crystalline phases.

### 3.4. Porosity

**Figure 9:**
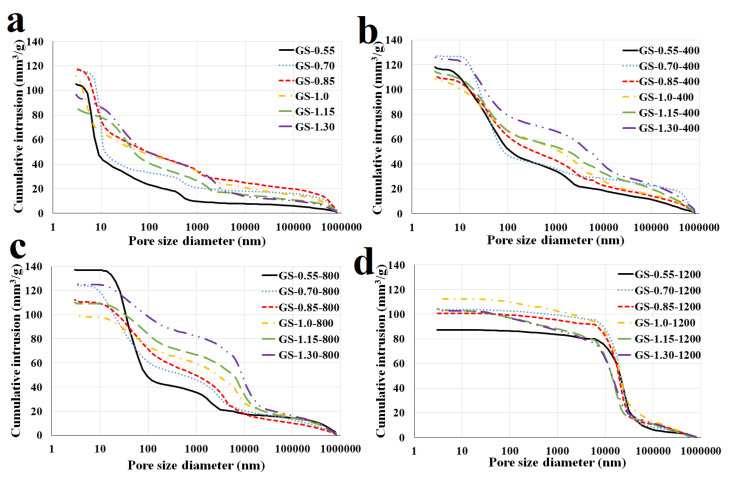
Mercury intrusion porosimetry of the geopolymer composites with various molar ratios of K/Al at laboratory temperature (**a**) and after exposure to 400 °C (**b**), 800 °C (**c**), and 1200 °C (**d**).

The pore size distributions of geopolymer composites before and after exposure to elevated temperatures of 400, 800, and 1200 °C were characterized by mercury intrusion porosimetry. As shown in Figure 9, characteristic pore size gradually increased with the increase of temperature, especially from a temperature of 800 °C. Geopolymer composites contained mainly mesopores up to 10 nm after curing at laboratory temperature (25 °C). Therefore, the average pore size increased from tens of nanometers to hundreds (samples GS-1.0, GS-1.15, and GS-1.30) or micrometers (samples GS-0.55, GS-0.70, and GS-0.85) after exposure to a temperature of 1200 °C. Pore volume of test samples increased slightly with increasing temperatures up to 800 °C (probably due to dehydroxylation) and then decreased at 1200 °C. Significant changes in porosity from 800 °C were probably caused by crystallization of the new phases which caused the shrinkage of the geopolymer matrix.

**Figure 10:**
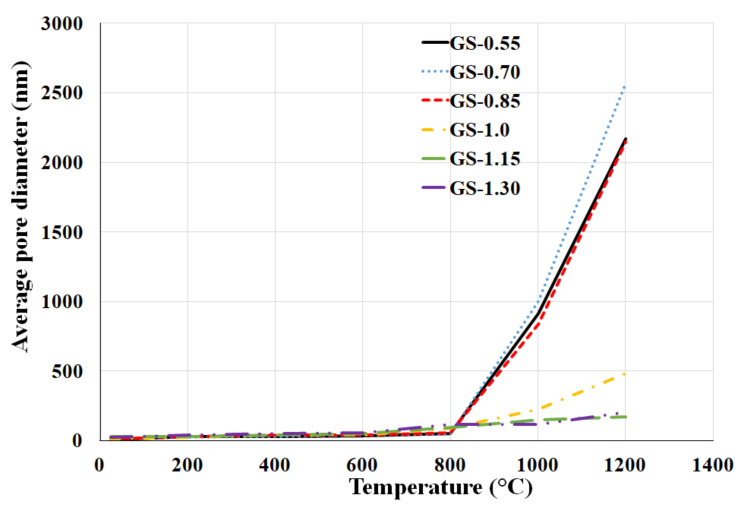
The influence of increasing temperature on the average pore diameter of geopolymer composites.

As shown in Figure 9 and Figure 10, from laboratory temperature (25 °C) to 800 °C, with the increasing of K/Al, the pore size more or less increased, while from 1000 °C to 1200 °C, the pore size drastically decreased with higher molar ratios. A large decrease occurred in the sample GS-1.0. Samples GS-0.55, GS-0.70, and GS-0.85 had values of average pore size 10, 14, and 15 nm after hardening at laboratory temperature and 2172, 2570, and 2145 nm after exposure to 1200 °C. On the contrary, samples GS-1.0, GS-1.15, and GS-1.30 had values of average pore size 11, 27, and 26 nm after hardening at laboratory temperature and 485, 170, and 206 nm after exposure to 1200 °C. The effect of K/Al on pore volume was not significant. Pore volume of all samples ranged from 90 to 120 mm^3^/g. Rovnaník et al. [1] investigated the porosity of matekaolin-based geopolymer composite filled with chamotte before and after exposure to high temperatures. They found out that values of pore volume were 120 mm^3^/g after exposure to high temperatures (up to 1000 °C).

### 3.5. Morphology

**Figure 11:**
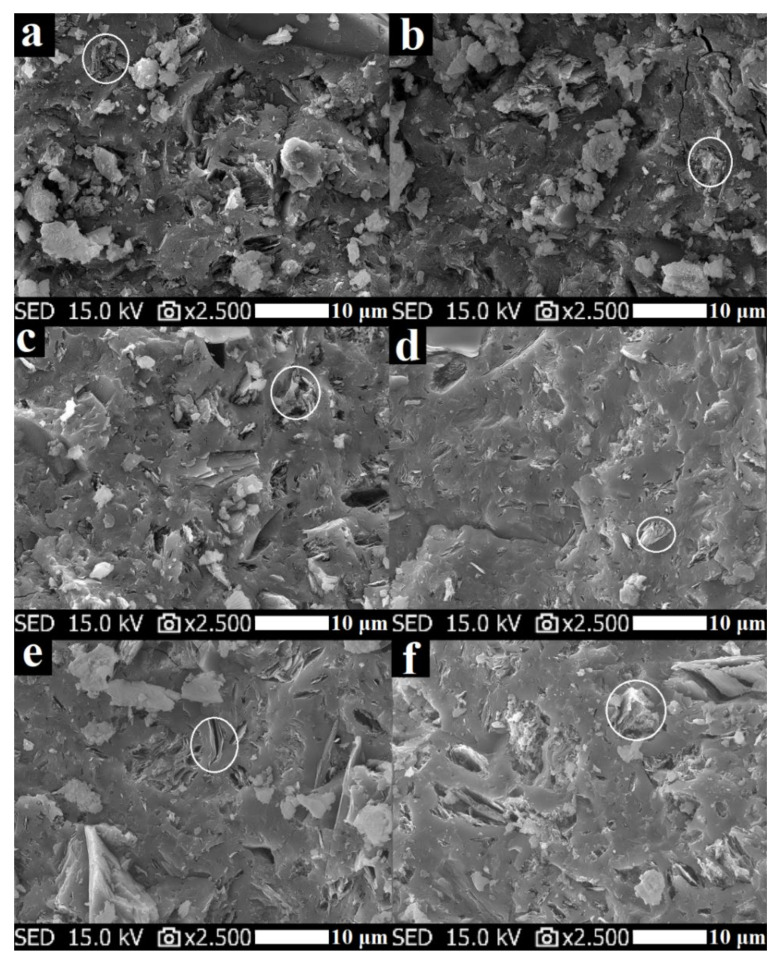
Micrographs of geopolymer binders with various molar ratios of K/Al ((**a**)—GB-0.55, (**b**)—GB-0.70, (**c**)—GB-0.85, (**d**)—GB-1.0, (**e**)—GB-1.15, (**f**)—GB-1.30) at laboratory temperature.

Figure 11 shows SEM images of geopolymer binders with various molar ratios of K/Al after hardening. The images showed that the geopolymer matrix contained undissolved plates of metakaolinite (highlighted by a white circle in Figure 11). The structure was mostly compact and did not contain a large number of visible pores. The exceptions were samples GB-0.55 and GB-0.70, which had a relatively loose gel with agglomerated fine particles and less platy particles. That is, the increase of potassium content is beneficial for geopolymerization. The increase in density with increasing molar ratios of Na/Al was also observed by Hou et al. [32].

**Figure 12:**
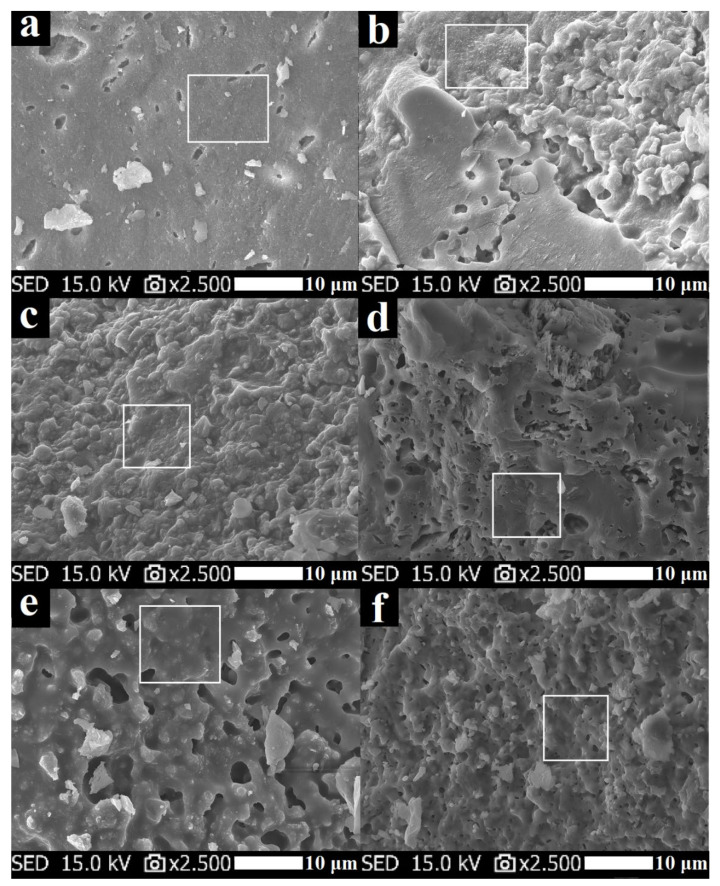
Micrographs of geopolymer binders with various molar ratios of K/Al ((**a**)—GB-0.55, (**b**)—GB-0.70, (**c**)—GB-0.85, (**d**)—GB-1.0, (**e**)—GB-1.15, (**f**)—GB-1.30) after exposure to 1000 °C.

SEM images of geopolymer binders with different K/Al molar ratios after exposure to elevated temperature (1000 °C) are shown in Figure 12. As can be seen, the structure of the geopolymer matrix rapidly changed after exposure to high temperature. The post exposure geopolymer binders had fewer inclusions and exhibited a smoother homogeneous texture than the unexposed geopolymer (highlighted by a white square in Figure 12). The structure was porous, which is consistent with the results of porosity. This was caused by the viscous sintering and phase changes of the geopolymer binders during high-temperature exposure. Similar findings about the structure of a geopolymer binder prepared from metakaolin after exposure to elevated temperatures were previously documented by Bell et al. [52], Lahoti et al. [29], and Duxson et al. [40].

## 4. Conclusions

In this study, the effect of the K/Al ratio at elevated temperatures on thermo-mechanical properties of metakaolinite-based geopolymers filled with chamotte was investigated. The following conclusions can be drawn from the obtained results:

- The X-ray diffractograms showed that geopolymers had a stable amorphous phase up to temperatures of 800–1200 °C depending on the K/Al ratio. The temperature of formation of the crystalline phases decreased with increasing K/Al ratios.
- Increasing of K/Al ratio led to a decline in the onset temperature of the major shrinkage. The addition of chamotte into geopolymer binders resulted in a decrease of total shrinkage of the geopolymer composites (from 12–20% to 0.4–2.1%).
- Increasing addition of potassium content led to a rise in the refractoriness of geopolymer binders. The addition of chamotte to geopolymer binders resulted in a reduction of their refractoriness.
- The compressive and flexural strength and modulus of elasticity decreased with growing K/Al ratios. The exception was the local maximum at K/Al ratio 1, which was the most evident at laboratory temperature and at 200 °C.
- Results of the compressive strength tested in situ did not show comparable results with compressive strength tested after exposure to elevated temperatures, which followed a decreasing path with increasing temperature. The values of compressive strength tested in situ began to rise from 600 °C.
- Average pore diameter increased slightly with increasing potassium content at laboratory temperature and at elevated temperatures up to 800 °C, while from 1000 °C to 1200 °C, the pore size drastically decreased. The effect of K/Al ratio on total pore volume was not significant.
- As a result of calcination at high temperatures, phase changes occurred in the binder and the inhomogeneous geopolymer matrix with undissolved metakaolinite plates turning into a homogeneous porous structure with visible pores.

The results of this study showed that geopolymer composites containing chamotte with K/Al ratios equal to one had optimal properties at laboratory temperature. Sample GS-1.0 displayed high mechanical strength at laboratory temperature. GS-1.0 can be used under load up to a temperature of approximately 600 °C, and at higher temperature the GS-1.0 becomes plastic under load, although heat resistance (without load) was 1500 °C. For thermal applications above 600 °C, it is better to use samples with lower K/Al ratios (sample 0.55 or 0.70), which have high refractoriness (approx. 1650 °C) and high mechanical strength after thermal exposure and even in situ under high temperatures. However, the samples with lower K/Al ratios have higher shrinkage during the first heating.

## Figures and Tables

**Table 1:** Chemical composition (wt%) of raw materials according to XRF measurements.

Material		Material Composition (%)											
	^a^ LOI	H_2_O	SiO_2_	Al_2_O_3_	Fe_2_O_3_	CaO	MgO	Na_2_O	K_2_O	TiO_2_	P_2_O_5_	V_2_O_5_	ZrO_2_
Mefisto L_05_	1.30	-	52.2	42.9	0.88	0.16	-	-	0.75	1.62	0.07	0.05	0.03
Potassium silicate	-	60.4	27.0	0.05	0.01	-	-	0.38	12.1	-	-	-	-
Chamotte	0.02	-	52.1	43.6	1.24	0.17	0.12	0.05	0.94	1.65	0.06	-	0.05

^a^ LOI = Loss on ignition.

**Table 2:** Physical properties of raw materials.

Material	Specific Gravity	Bulk Density	Particle Size		Specific Surface Area (BET)
	(kg/m^3^)	(kg/m^3^)	d50 (µm)	d90 (µm)	(m^2^/g)
Mefisto L_05_	2455	614	5.58	16.47	13.4
Chamotte	2541	1494	-	-	-

## Data Availability

The data presented in this study are available on request from the corresponding author.
